# Ergosterol Ameliorates Diabetic Nephropathy by Attenuating Mesangial Cell Proliferation and Extracellular Matrix Deposition via the TGF-β1/Smad2 Signaling Pathway

**DOI:** 10.3390/nu11020483

**Published:** 2019-02-25

**Authors:** Zhonghua Dong, Yueyue Sun, Guangwei Wei, Siying Li, Zhongxi Zhao

**Affiliations:** 1 School of Pharmaceutical Sciences, Shandong University, 44 West Wenhua Road, Jinan 250012, Shandong, China; 201514330@mail.sdu.edu.cn (Z.D.); 13021717075@163.com (Y.S.); 2 School of Basic Medical Sciences, Shandong University, 44 West Wenhua Road, Jinan 250012, Shandong, China; gwwei@yahoo.com (G.W.); lisiying@sdu.edu.cn (S.L.); 3 Shandong Engineering & Technology Research Center for Jujube Food and Drug, 44 West Wenhua Road, Jinan 250012, Shandong, China

**Keywords:** ergosterol, diabetic nephropathy, mesangial cells, ECM, TGF-β1

## Abstract

(1) Background: Diabetic nephropathy, a microvascular complication of diabetes, is one of the principal causes of end-stage renal disease worldwide. The aim of this study was to explore the therapeutic effects of ergosterol on diabetic nephropathy. (2) Methods: Streptozotocin (STZ)-induced C57BL/6 diabetic mice were treated with ergosterol (10, 20, 40 mg/kg/day) for 8 weeks by oral gavage. The in vitro study employed rat mesangial cells exposed to 30 mM glucose for 48 h in the presence of 10 or 20 μM ergosterol. (3) Results: Ergosterol treatment improved body weights, ameliorated the majority of biochemical and renal functional parameters and histopathological changes, and reduced extracellular matrix (ECM) deposition in diabetic mice. In vitro, ergosterol suppressed proliferation, reduced the levels of ECM proteins, and increased the expression of matrix metalloproteinase-2 and -9 in high glucose-induced mesangial cells; Furthermore, ergosterol markedly improved transforming growth factor-β1 (TGF-β1) expression, enhanced phosphorylation levels of drosophila mothers against decapentaplegic 2 (Smad2), and regulated the downstream factors in vivo and in vitro. (4) Conclusions: Ergosterol alleviated mesangial cell proliferation and the subsequent ECM deposition by regulating the TGF-β1/Smad2 signaling pathway.

## 1. Introduction

Diabetes mellitus (DM) is a chronic condition that occurs when there are raised blood glucose levels, arises from pancreas’ failure to secret enough insulin and/or decreased response to insulin [1]. Diabetic nephropathy, one of the most serious long-term complications of DM, has been considered the leading cause of end-stage renal disease (ESRD) [2]. It is characterized by proteinuria, an expanded mesangial area, a thickening of basement membrane, persistent renal fibrosis, and progressive renal function decline, which are caused by the accumulation of extracellular matrix (ECM) deposition [3]. Currently there are no effective therapies available, so it is urgent to search for more effective drugs to delay the progression of diabetic nephropathy (DN) [4].

Glomerular mesangial cells maintain the structural architecture of the glomerular capillary and mesangial matrix homeostasis as well as regulating the filtration surface area [5]. These cells can be stimulated by high ambient glucose and undergo over-proliferation, hypertrophy, and increasing synthesis of extracellular matrix proteins [5,6]. The excessive accumulation of ECM components in the glomerulus results in glomerulosclerosis and contributes to the occurrence and development of DN. The deposition of ECM during the progression of DN may due to the increased synthesis and decreased degradation [7,8]. Matrix metalloproteinases (MMPs) are reported to be involved in the breakdown and turnover of ECM, indicating that the altered MMP production may be a potential approach to prevent DN [9]. Transforming growth factor-β1 (TGF-β1) is a multifunctional cytokine that is involved in the development of glomerulosclerosis and interstitial fibrosis [10]. Hyperglycemia increases the expression of TGF-β1, which enhances the deposition of ECM proteins such as fibronectin and collagens and inhibits the activity of MMPs, thus eliciting mesangial expansion and glomerular basement membrane thickening [11,12]. Therefore, inhibiting the proliferation of mesangial cells and ECM protein production may be an effective way to ameliorate diabetic nephropathy.

**Figure 1:**
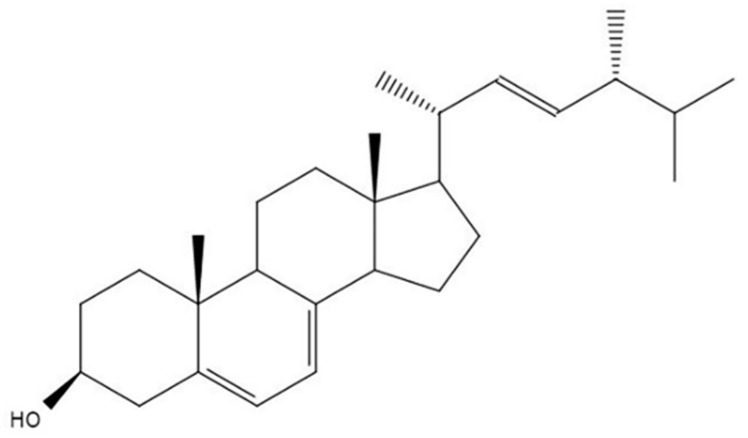
Structure of ergosterol.

In recent years, research on phytosterols has become an emerging field, significant potential of phytosterols has been found for the prevention and treatment of various diseases and disorders. Phytosterols, a group of natural bioactive products, shows multiple pharmacological effects, such as reducing blood cholesterol and protecting against cardiovascular disease [13,14,15,16]. Ergosterol (Figure 1), one of the members of the phytosterol family found in edible fungi, is a provitamin form of vitamin D2 and plays a principal role in maintaining the integrity of fungal cell membranes [17]. Ergosterol has been linked to several pharmacological functions, such as anti-inflammatory [18], anti-oxidative [19], and anti-diabetic [20] activity, as well as ameliorating chronic obstructive pulmonary disease [21].

In this work, we examined reno-protective potentials of ergosterol in high glucose-exposed rat mesangial cells and in streptozotocin (STZ)-induced diabetic mice. In vitro, the therapeutic effects of ergosterol on the cell proliferation and extracellular matrix proteins were explored in the mesangial cells induced by high glucose. In the in vivo study, we explored the ameliorating effects of ergosterol on biochemical and renal functional parameters and histopathological changes in diabetic mice. These studies could provide further understanding of ameliorating effects of ergosterol on diabetic nephrotoxicity as well as paving a foundation for selecting the optimal reno-therapeutic agents containing ergosterol.

## 2. Materials and Methods

### 2.1. Chemicals

Dulbecco’s modified Eagle medium (DMEM), fetal bovine serum (FBS), and penicillin–streptomycin were obtained from Gibco Invitrogen (Carlsbad, CA, USA). D-glucose and D-mannitol were purchased from Sigma Chemical (St. Louis, MO, USA). 3-(4,5-Dimetylthiazol-yl)-diphenyl tetrazolium bromide (MTT) was supplied by Solarbio Technology Co. Ltd. (Beijing, China). Ergosterol, enalaprilat, and streptozotocin were obtained from Aladdin Biochemical Technology CO. Ltd. (Shanghai, China). A blood glucose meter and blood glucose test strips were provided by F. Hoffmann-La Roche Ltd. (Basel, Switzerland). A mouse insulin ELISA kit and mouse C-peptide ELISA kit were obtained from Cusabio Technology (Wuhan, China). A mouse albumin ELISA kit was purchased from Bethyl Laboratories (Montgomery, TX, USA). Creatinine, urea nitrogen, and total cholesterol assay kits were provided by the Nanjing Jiancheng Bioengineering Institute (Nanjing, China). LY2109761 (inhibitor of Smad2) was purchased from Selleck Chemicals (Munich, Germany). Proliferating cell nuclear antigen (PCNA), fibronectin (FN), MMP-2, MMP-9, and TGF-β1 antibodies were obtained from Abcam (Cambridge, UK). P-Smad2, Smad2, Smad4, and β-actin antibodies were purchased from Cell Signaling Technology (MA, USA). Collagen I alpha 1 and Smad7 and antibodies were provided by R&D system (Minneapolis, MN, USA). Goat anti-mouse and goat anti-rabbit immunoglobulin G (IgG) were obtained from ZSGB Bio-technology Co. Ltd. (Beijing, China). All the other reagents used in the experiments were analytical grade.

### 2.2. Cell Culture and Treatment

Rat mesangial cells (RMCs, American Type Culture Collection, Manassas, VA) were cultured with DMEM (low glucose) media, containing 10% FBS and 1% penicillin–streptomycin at 37 °C in 5% CO_2_. RMCs were cultured for 24 h in DMEM medium containing 5.6 mM glucose and 0.5% FBS and then used for experiments. Cells were cultured with D-glucose at normal (5.6 mM) or high (30 mM) concentrations in DMEM medium. D-Mannitol (24.4 mM) was added along with 5.6 mM glucose as a control of osmolality for 48 h. Ergosterol was dissolved in dimethyl sulfoxide (DMSO) and freshly diluted with culture medium for live culture with cells; a final culture concentration of DMSO was <0.1%. RMCs cultured with 30 mM glucose were co-treated with or without ergosterol (10, 20 μM).

### 2.3. Cell Proliferation Analysis

The effect of ergosterol on cell proliferation was measured by MTT assay. RMCs were seeded in 96-well culture plates at 4 × 10^3^ cells per well and incubated in DMEM (low glucose) containing 10% FBS. After 48 h incubation under different conditions as described above, 20 μL MTT (5 mg/mL) were added to each well and incubation continued at 37 °C for 4 h. Then, 150 μL of DMSO were added into each well to dissolve the formazan crystals. The optical density (OD) value of each well was measured at the wavelength of 570 nm. The arithmetic mean OD of six wells for each group was calculated and these results were expressed as the percentage of cell viability compared with cells in control group.

### 2.4. Animal Models

The study was conducted in accordance with the Declaration of Helsinki, and the protocol was approved by the Animal Care and Use Committee of Shandong University (No. 2016020, Jinan, Shandong, China). Male C57BL/6 mice of age six weeks and weight 18–20 g were purchased from the Laboratory Animal Center of Shandong University (Jinan, Shandong, China). All mice were housed under controlled temperature (22 ± 2 °C), in 50–60% relative humidity, with a 12-h day/night cycle, and had free access to food and water. After one week of adaptation and following 16-h overnight fasting, the mice were induced to diabetes by intraperitoneal injection of freshly prepared STZ (dissolved in 0.01 M citrate buffer, pH 4.5) at a single dose of 130 mg/kg. At the same time, the normal control group was injected with citrate-phosphate buffer. Seventy-two hours after STZ injection, blood glucose levels were measured. Mice with blood glucose concentration over 16.7 mM were classified as a successful diabetes model and used in the study. Experimental mice were randomized into seven groups (*n* = 6 per group): the normal control group (NC group), the diabetic nephropathy group (DN group), the ergosterol-treated group (40 mg/kg/day, NC + ERG group), the ergosterol-treated diabetic groups (10, 20 or 40 mg/kg/day, DN+ERG group), and the enalaprilat-treated diabetic group (1.5 mg/kg/day, DN+ENA group). ERG was dissolved in 0.5% sodium carboxymethyl cellulose (CMC-Na) and administered to mice by oral gavage at a dose volume of 0.1 mL per 10 g body weight, whereas the mice in the NC and DN group received 0.5% CMC-Na aqueous solution. Seven days after STZ injection, mice in all groups were received intragastric administration once per day for eight consecutive weeks. All mice had free access to food and water during the experimental period.

The body weights were monitored once a week and fasting blood glucose levels was measured every 2 weeks using the blood drawn from a tail vein by a blood glucose meter. At the end of the study, 24-h urine samples were collected from all mice using metabolic cages for the measure of 24-h urine volume and urinary albuminuria (Alb). Blood samples were drawn from the orbits of all mice and centrifuged at 3000 g (15 min, 4 °C) after clotting. Serum insulin, C-peptide, serum creatinine (SCR), blood urea nitrogen (BUN), and total cholesterol (TC) levels of the mice were evaluated by assay kits. Then, the mice were sacrificed, and their kidney tissues were immediately removed. The kidney index was calculated as the ratio of kidney-weight-to-body-weight. The left kidney was placed in liquid nitrogen and stored at −80 °C for biochemical analysis, the other one was fixed with 10% paraformaldehyde for paraffin sectioning.

### 2.5. Histological and Morphological Examination

The right kidney samples were washed with phosphate buffered solution (PBS) and peeled off the kidney capsule. After that, the kidney tissues were fixed in 10% buffered formaldehyde solution, embedded in paraffin. The paraffin sections of 4-μm thickness were then stained with hematoxylin and eosin (HE), periodic acid-Schiff (PAS) and Masson’s trichrome for routine renal histopathological examination and the visualization of glycogen and collagen fibers by a morphometric microscope (Olympus Corporation, Tokyo, Japan) at 400× magnification. Slides were assessed in a blind manner. Twenty-five glomeruli were randomly selected from each section; PAS-positive areas and glomerular volumes were examined using Image-Pro Plus 6.0 software (Media Cybernetics, Silver Spring, MD, USA). The glomerular volume was calculated using the formula: Glomerular Volume = glomerular area^1.5^ × 1.38/1.01 [22]. The fibrosis level was assessed by calculating the percentage area of blue staining component in the section of Masson’s trichrome staining using Image-Pro Plus 6.0 software.

### 2.6. Immunohistochemical Analysis

For immunohistochemical analysis, mouse kidney paraffin sections were deparaffinized, rehydrated, and subjected to microwave-based antigen retrieval in citrate buffer. The sections were blocked for 20 min with 10% normal goat serum after 3 times washing with PBS. The kidney sections were incubated overnight with primary anti-fibronectin and collagen I antibodies at 4 °C. A secondary biotinylated antibody was added onto the sections at 37 °C for 30 min. Immunostaining was visualized using 3,3-diaminobenzidine (DAB, ZSGB-BIO). Images of fibronectin and collagen I (Col I) were obtained and photographed under a microscope (Olympus Corporation, Tokyo, Japan) at 200 × magnifications.

### 2.7. Western Blot Analysis

For protein preparation, the kidneys and rat mesangial cells were lysed in Radio Immunoprecipitation Assay (RIPA) lysis buffer (Millipore, Bedford, MA, USA) respectively. Samples containing 30 μg total protein were loaded into 6–10% SDS-PAGE and transferred onto polyvinylidene difluoride (PVDF) membranes (Millipore, Bedford, MA, USA). Subsequently, the membranes were blocked in TBST containing 5% (*w/v*) non-fat milk for 2 h at room temperature, and then incubated overnight with the primary antibodies (fibronectin, collagen I, MMP-2, MMP-9, TGF-β1, p-Smad2, Smad2, Smad4, Smad7, 1:1000; anti-β-actin, 1:5000) at 4 °C. The membranes were washed three times with TBST and incubated with a secondary antibody of goat anti-rabbit IgG or goat anti-mouse IgG conjugated to horseradish peroxidase diluted 1:5000 in the blocking buffer for 2 h. The protein bands were visualized with the chemiluminescence (ECL) detection system (Amersham, Little Chalfont, UK) and quantified by AlphaView SA software.

### 2.8. Quantitative Real-Time RT-PCR

Total RNA was extracted from mouse kidney tissues using TRIzol^®^ reagent (Life Technologies, Inc., MD, USA) and reverse-transcribed using a first-strand cDNA kit (Toyobo, Shanghai, China) according to the manufacturer’s protocol. Real-time quantitative polymerase chain reaction (PCR) of samples was performed using the specific primers, SYBR Green I reagent and the RT-PCR kit on Bio-Rad iQ5 Quantitative PCR System (Takara, China). Relative mRNA levels were calculated using the 2^−ΔΔCt^ method and normalized to the expression of β-actin. The primer sequences are provided in Table 1.

### 2.9. Statistical Analysis

All data were presented as the means ± SD (standard deviation) of at least three experiments. The Student’s *t*-test and ANOVA were performed between different groups using GraphPad Prism. Data were considered statistically significant for *p* < 0.05.

## 3. Results

### 3.1. Effects of Ergosterol on Metabolic and Biochemical Parameters

**Figure 2:**
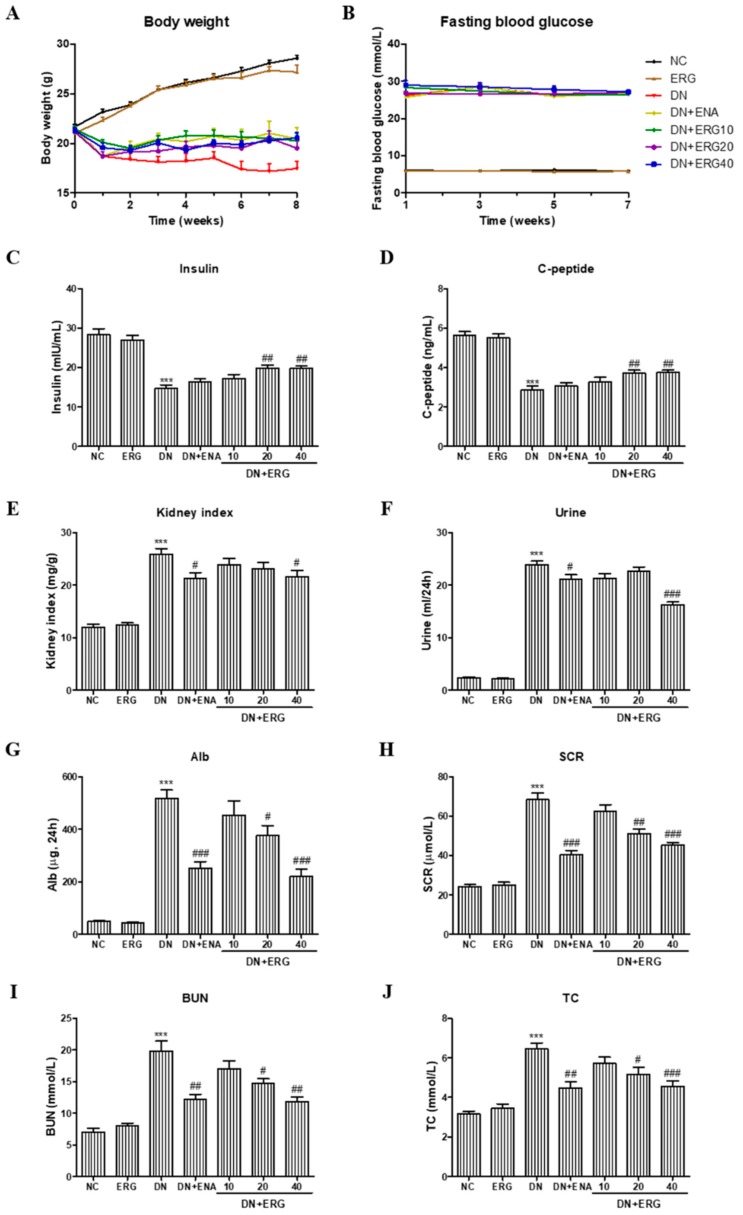
Effects of ergosterol on body weight, fasting blood glucose, serum insulin, C-peptide, and renal functional parameters in streptozotocin (STZ)-induced diabetic mice. (**A**) Body weight, (**B**) fasting blood glucose, (**C**) serum insulin, (**D**) C-peptide, (**E**) kidney index, (**F**) 24-h urine volume, (**G**) Alb, (**H**) SCR, (**I**) BUN, and (**J**) TC. Alb: 24-h urinary albuminuria; SCR: serum creatinine; BUN: blood urea nitrogen; TC: total cholesterol; NC: normal control; DN: diabetic nephropathy model induced by STZ; ENA: positive control, 1.5 mg/kg/day enalaprilat administration; ERG10, ERG20, ERG40: 10, 20, 40 mg/kg/day ergosterol administration, respectively. Data are presented as the means ± SD, *n* = 6. *** *p* < 0.001 compared with the NC group; # *p* < 0.05, ## *p* < 0.01, ### *p* < 0.001 compared with the DN group.

Body weights and fasting blood glucose were monitored during the experiments. Compared with the age-matched normal mice, the body weight of mice in DN model group gradually decreased. While, long-term treatment (8 weeks) with low-dose and high-dose ergosterol could attenuate the weight loss of the DN mice (Figure 2A). The fasting blood glucose of mice in DN group increased significantly after the injection of STZ. Ergosterol administration had no distinguishable hypoglycemic effect over the 8 weeks of treatment (Figure 2B). However, the levels of serum insulin and C-peptide in STZ-induced diabetic mice were notably decreased compared with the normal control group and were significantly improved by the administration of ERG (Figure 2C,D), indicating that the treatment of ERG may ameliorate the endocrine function of pancreatic β-cells.

The effects of ergosterol on kidney index and renal functional parameters in STZ-induced diabetic mice were also examined. Hypernephrotrophy was found in the DN group assessed by an increase of kidney index but was significantly reduced by treatment with high-dose ergosterol (Figure 2E). Furthermore, compared with the mice in the normal group, the levels of 24-h urine volume, 24-h urinary albuminuria, SCR, BUN, and TC in diabetic mice were significantly increased. However, with ergosterol and the positive control drug there was a prominent decrease in these renal functional parameters (Figure 2F–J).

### 3.2. Effects of Ergosterol on Histopathological Changes in Renal Tissue

**Figure 3:**
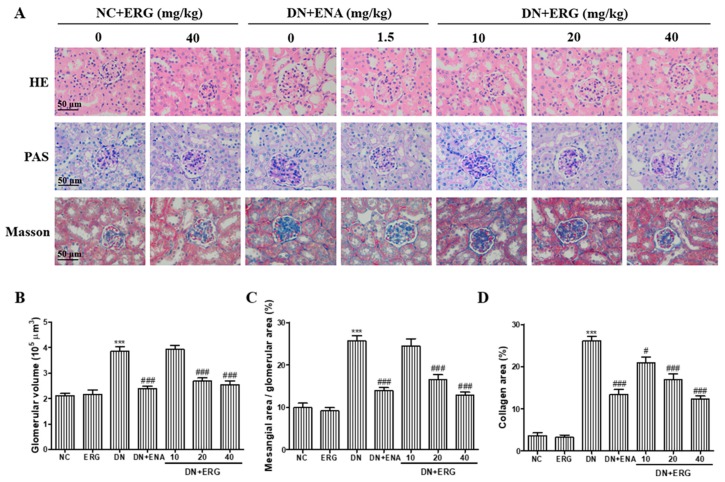
Effects of ergosterol on histopathological changes in the renal tissues of STZ-induced diabetic mice. (**A**) Representative hematoxylin-eosin (HE), periodic acid-Schiff (PAS) and Masson staining in kidneys from formalin-fixed kidney sections taken from the mice of each group (*n* = 6/group) at 8 weeks. (**B**–**D**) Quantitative assessment of glomerular volume, mesangial area (%), and collagen area (%). NC: normal control; DN: diabetic nephropathy; ENA: enalaprilat administration; ERG: ergosterol administration. *** *p* < 0.001 compared with NC; # *p* < 0.05, ### *p* < 0.001 compared with DN.

After eight weeks of treatment with ergosterol, histological examination of kidneys from all seven groups was performed through HE, PAS staining (Figure 3A). The glomerular hypertrophy was measured by HE staining (Figure 3B). The lumen of renal tubules was unnaturally widened, the epithelial cells were gravely damaged, and the renal tubule appeared partial tubular epithelial vacuole degeneration. For the DN mice given ergosterol or enalaprilat, the degree of pathological changes was significantly attenuated. Mesangial matrix expansion was measured by PAS staining. Marked mesangial expansion and glycogen storage were observed in DN mice, which were significantly attenuated by the treatment with medium- and high-dose ergosterol (Figure 3C). In addition, there were no notable changes in renal histology in ergosterol-treated normal mice. According to these results from Masson’s staining, STZ challenge led to a marked deposition of collagen, whereas ergosterol administration significantly relieved the deposition of collagen fiber (Figure 3D).

### 3.3. Ergosterol Significantly Inhibited the Proliferation of Renal Mesangial Cells

**Figure 4:**
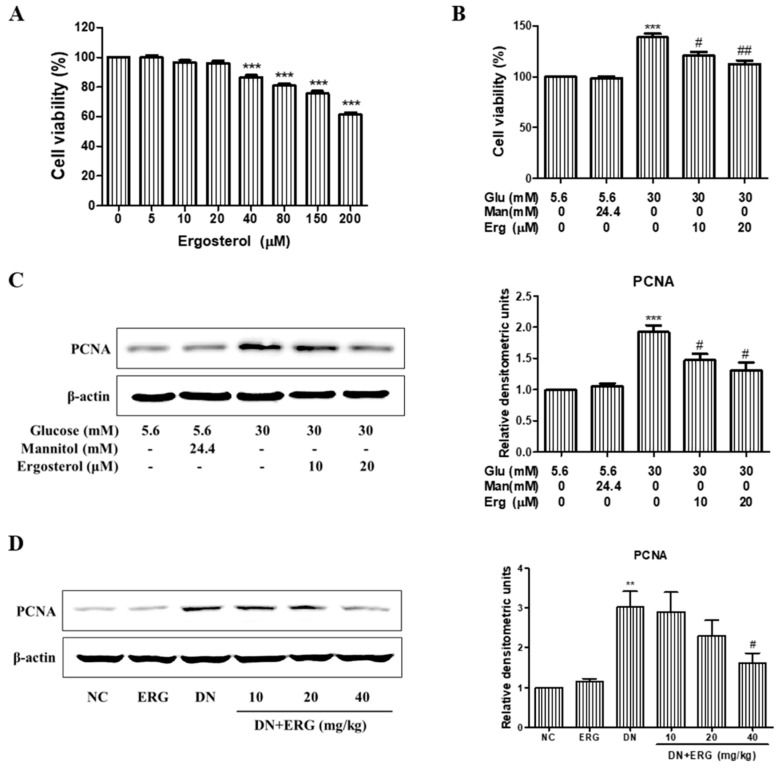
Ergosterol inhibited the proliferation of renal mesangial cells. (**A**) Rat glomerular mesangial cells (RMCs) were co-treated with and without ergosterol (10 or 20 μM) under normal glucose conditions (5.6 mM glucose) for 48 h. Cell proliferation was measured by the 3-(4,5-dimetylthiazol-yl)-diphenyl tetrazolium bromide (MTT) assay. (**B**) RMCs were co-treated with and without ergosterol (10 or 20 μM) under high glucose conditions (30 mM glucose) for 48 h. Cell proliferation was evaluated by the MTT assay. (**C**,**D**) Western blot analysis of proliferating cell nuclear antigen (PCNA) expression in cultured RMCs (Data are presented as the means ± SD, triplicate) and renal parenchyma (data are presented as the means ± SD, *n* = 3). NC: normal control; DN: diabetic nephropathy model induced by STZ; ERG: ergosterol administration. ** *p* < 0.01, *** *p* < 0.001 compared with normal glucose or normal control; # *p* < 0.05, ## *p* < 0.01 compared with high glucose or diabetic nephropathy model.

Increased proliferation of mesangial cells contributes to the mesangial hypertrophy and glomerular fibrosis. Thus, we investigated the effects of ergosterol on high glucose induced mesangial cell proliferation by MTT assay. As is shown in Figure 4A, the proliferation of RMCs was not altered by ergosterol treatment under normal glucose. Meanwhile, we observed that high glucose significantly promoted RMC proliferation compared with normal glucose group. However, the proliferative capacity of RMCs treated with ergosterol significantly decreased in a dose-dependent manner under high glucose conditions (Figure 4B). The expression levels of PCNA were determined by Western blot. With comparison to the control group, the protein levels of PCNA were significantly amplified in high glucose group. Whereas, co-treatment with ergosterol downregulate the expression of PCNA in high glucose-induced RMCs (Figure 4C). Likewise, ergosterol prominently inhibited PCNA expression in the renal parenchyma of STZ-induced diabetic mice (Figure 4D).

### 3.4. Ergosterol Significantly Alleviated ECM Accumulation

**Figure 5:**
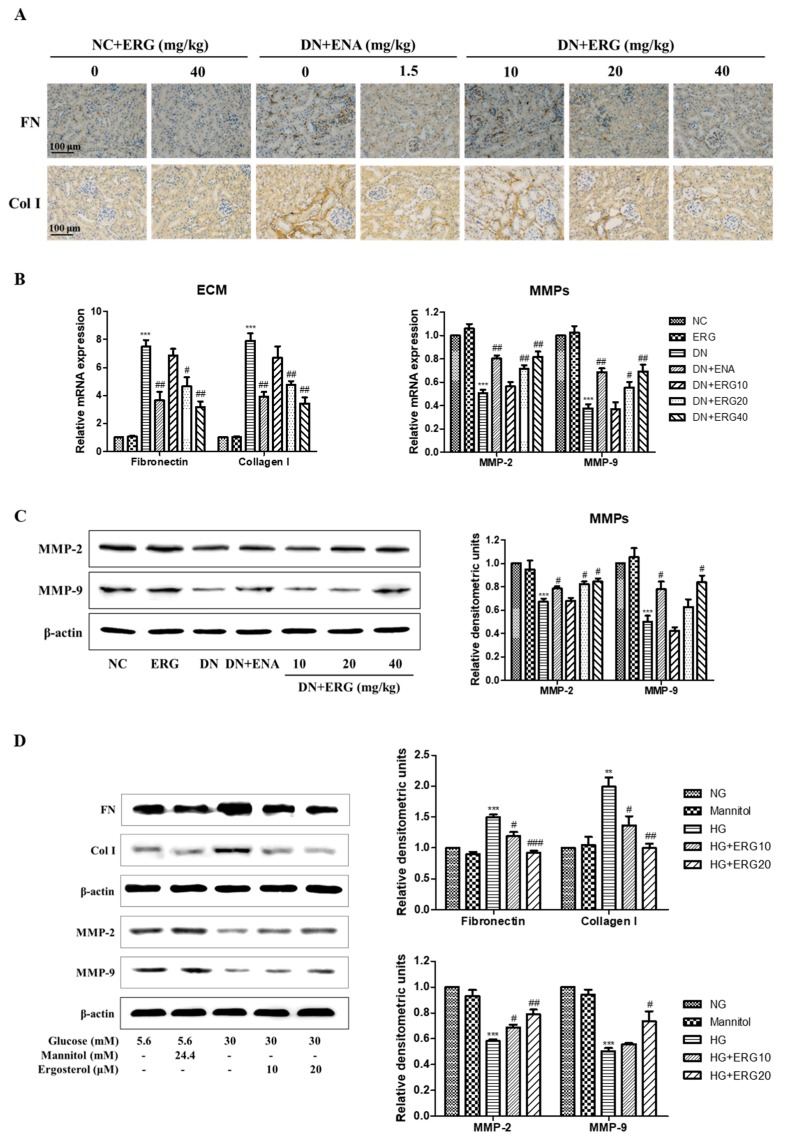
Ergosterol alleviated extracellular matrix (ECM) accumulation. (**A**) Representative renal sections with immunohistochemical staining of fibronectin and collagen I. (**B**) Expression of fibronectin, collagen I, MMP-2, and MMP-9 mRNA levels in renal parenchyma (data are presented as the means ± SD, *n* = 3). (**C**) Expression of MMP-2 and MMP-9 protein levels in renal parenchyma through Western blotting (data are presented as the means ± SD, *n* = 3). (**D**) Expression levels of fibronectin, collagen I, MMP-2 and MMP-9 in RMCs cultured under different conditions through Western blotting (data are presented as the means ± SD, triplicate). NC: normal control; DN: diabetic nephropathy model induced by STZ; ENA: positive control, 1.5 mg/kg/day enalaprilat administration; ERG10, ERG20, ERG40: 10, 20, 40 mg/kg/day ergosterol administration, respectively. NG: normal glucose, 5.6 mM glucose; Mannitol: 5.6 mM glucose + 24.4 mM mannitol; HG: high glucose, 30 mM glucose; ERG10 and ERG 20: 10 and 20 μM ergosterol, respectively. ** *p* < 0.01, *** *p* < 0.001 compared with NG or NC; # *p* < 0.05, ## *p* < 0.01, ### *p* < 0.001 compared with HG or DN.

The accumulation of ECM proteins is a main hallmark for DN while fibronectin and collagen I are important compositions of ECM of renal interstitial fibrosis. The expression of fibronectin and collagen I proteins was evaluated after 8-week ergosterol treatment in the diabetic mice through immunohistochemistry and RT-PCR analysis. Figure 5A,B). Compared with the normal control group, both fibronectin and collagen I protein were abundant expressed in the diabetic nephropathy group, but markedly reduced in ergosterol-supplemented diabetic nephropathy mice (Figure 5A). Similarly, RT-PCR data shows that the relative mRNA expression of fibronectin and collagen I was reduced in ergosterol-treated diabetic mice (Figure 5B).

MMP-2 and MMP-9 catalyze the degradation of ECM components. To explore whether MMPs are involved in the beneficial effects of ergosterol on ECM accumulation, we monitored the mRNA and protein expressions of MMP-2 and MMP-9 in the diabetic kidney. Compared with the normal control group, the mRNA expression of MMP-2 and MMP-9 was significantly reduced in diabetic nephropathy group. However, ergosterol treatment efficiently reversed the reduction of MMP-2 and MMP-9 in diabetic kidneys (Figure 5B). Similar results were observed in Western blotting data (Figure 5C).

Next, we measured the effect of ergosterol on ECM proteins expression in RMCs induced by high glucose. In vitro studies show similar results to those in mice (Figure 5D). These results of Western blot analysis demonstrate that the expression levels of fibronectin and collagen I were markedly increased and the MMP-2 and MMP-9 levels were significantly reduced in high glucose-induced RMCs. However, treatment with ergosterol effectively abrogated the high-glucose (HG)-induced changes of these proteins’ expression.

### 3.5. Ergosterol Inhibited Renal Mesangial Cells Proliferation and ECM Accumulation through TGF-B1/Smad2 Pathway

**Figure 6:**
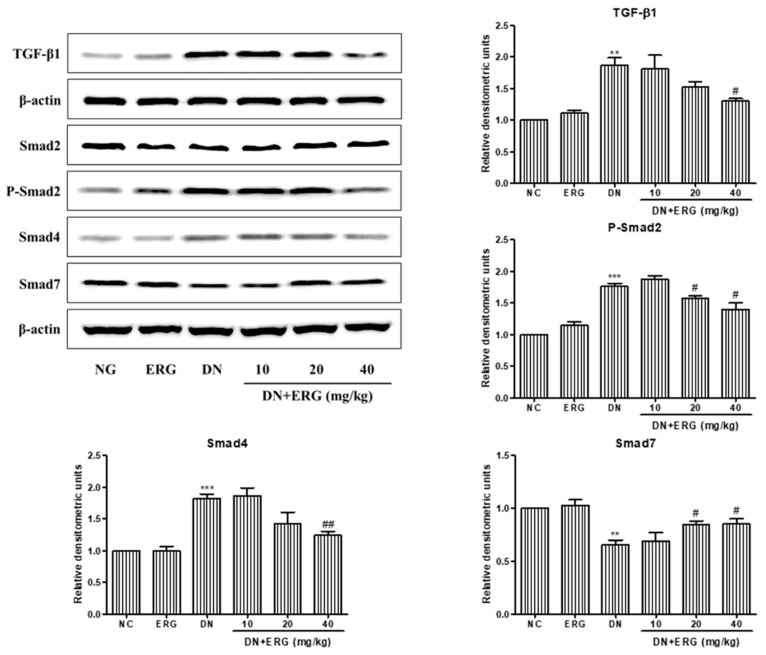
Ergosterol inhibited the renal transforming growth factor-β1 (TGF-β1)/ drosophila mothers against decapentaplegic 2 (Smad2) signaling pathway in STZ-induced diabetic mice. This figure shows these results of Western blot analysis of TGF-β1, Smad2, phosphor-Smad2, Smad4, and Smad7. NC: normal control; DN: diabetic nephropathy model induced by STZ; ERG10, ERG20, ERG40: 10, 20, 40 mg/kg/day ergosterol administration. Data are presented as the means ± SD, *n* = 3. ** *p* < 0.01, *** *p* < 0.001 compared with NC; # *p* < 0.05, ## *p* < 0.001 compared with DN.

**Figure 7:**
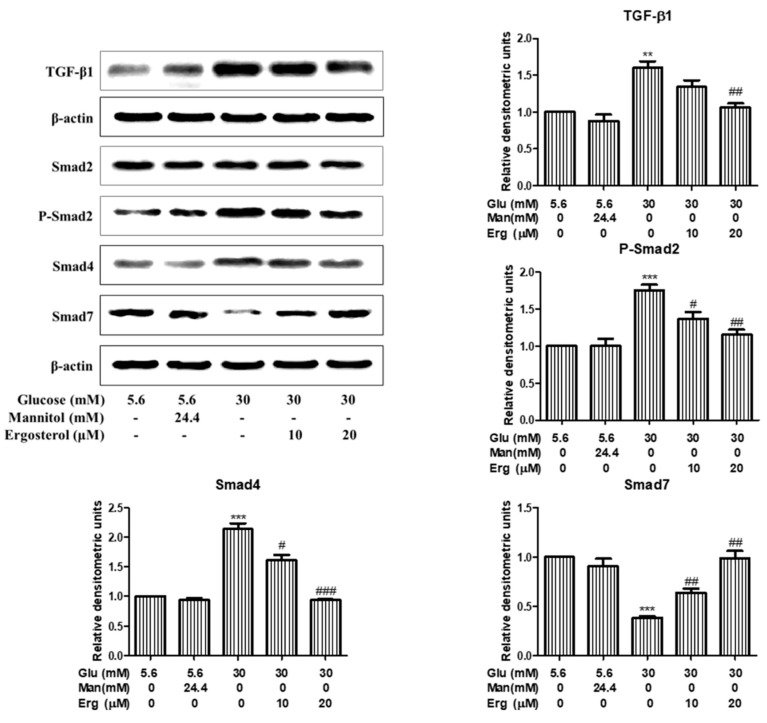
Ergosterol inhibited the TGF-β1/Smad2 signaling pathway in RMCs exposed to high glucose. This figure shows the expression levels of TGF-β1, Smad2, phosphorated-Smad2, Smad4, and Smad7 in RMCs cultured under different conditions through Western blotting. Glu: glucose; Man: mannitol; Erg: ergosterol. Data are presented as the means ± SD, performed in triplicate. ** *p* < 0.01, *** *p* < 0.001 compared with normal glucose; # *p* < 0.05, ## *p* < 0.01, ### *p* < 0.001 compared with high glucose.

Previous studies have confirmed that TGF-β1/Smads signaling plays a critical role in the development of DN, therefore, the change of TGF-β1/Smads pathway in the kidney was detected [23]. Western blot results indicate that the level of TGF-β1, phosphor-Smad2, and Smad4 in diabetic nephropathy group were significantly elevated compared to normal control group, while the level of Smad7 was significantly decreased, suggesting that the TGF-β1/Smad2 signaling pathway was activated in the diabetic nephropathy group (Figure 6). Strikingly, the protein levels of TGF-β1, phosphor-Smad2, Smad4, and Smad7 were reversed after 8-weeks treatment with ergosterol (Figure 6), indicating that ergosterol can inhibit the renal TGF-β1/Smad2 signaling pathway in STZ-induced diabetic nephropathy mice. Similar results were observed in ergosterol-treated RMCs (Figure 7).

**Figure 8:**
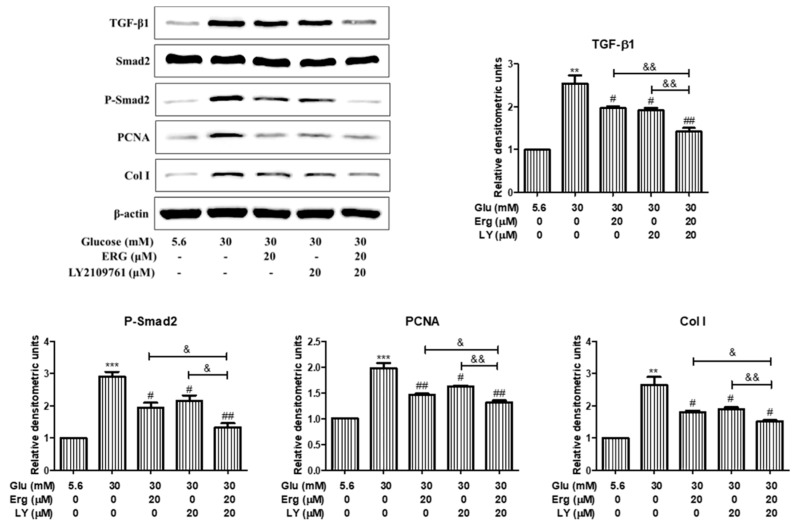
TGF-β1/Smad2 signaling was involved in the effects of ergosterol on high glucose-exposed RMCs. This figure shows the expression levels of TGF-β1, Smad2, phosphor-Smad2, PCNA, and collagen I in RMCs cultured under different conditions through Western blotting. Glu: glucose; Erg: ergosterol; LY: LY2109761, inhibitor of Smad2. Data are presented as the means ± SD, performed in triplicate. ** *p* < 0.01, *** *p* < 0.001 compared with the normal glucose; # *p* < 0.05, ## *p* < 0.01 compared with the high glucose; & *p* < 0.05, && *p* < 0.01 compared with the treatment of LY2109761 plus ergosterol.

To investigate whether the TGF-β1/Smad2 pathway was involved in the effect of ergosterol on mesangial cell proliferation and ECM expression, mesangial cells were pre-incubated with the inhibitor of Smad2 (LY2109761, 20 μM) for 30 min followed by the treatment with ergosterol (20 μM) for 48 h and the protein levels of PCNA and collagen I were measured. As is shown in Figure 8, after ergosterol or LY2109761 treatment, PCNA and collagen I expression were significantly inhibited compared with the high-glucose group, and these protein levels in cells treated with LY2109761 plus ergosterol were lower than cells treated with ergosterol or LY2109761 alone. These results infer that the effect of ergosterol on mesangial cell proliferation and ECM expression was associated with the suppression of TGF-β1/Smad2 signaling.

## 4. Discussion

DN is one of the most common and serious microvascular complications of DM and is the leading cause of ESRD. DN is characterized by marked mesangial matrix expansion resulting from hyperglycemia-induced stress and renal interstitial fibrosis resulting from ECM deposition, which lead to irreversible damage to kidney function [24,25]. However, the underlying mechanisms in the progression of DN are incompletely resolved, and there is no specific medicine for the treatment of DN, making DN a global burden. As previously reported, ERG exerts protective effects on diabetic nephropathy in STZ-injected mice [26]. However, the effects of ERG on histopathological changes and ECM deposition are not fully studied; this work was undertaken to assess the possible effects of ERG on DN.

Glucose metabolic dysfunction was caused by STZ injection, as evidenced by the increased glucose level as well as decreased serum insulin and C-peptide concentration. Although fasting blood glucose levels were not diacritically altered by the treatment of ERG, levels of serum insulin and C-peptide in ERG-treated diabetic mice were significantly increased. These results indicate that the treatment of ERG could improve, at least to a degree, the abnormality of glucose metabolism in STZ-induced diabetic mice.

During the process of DN, abnormal changes of renal functional parameters were observed from a macroscopic viewpoint, through significant increases in kidney index, urine volume, albuminuria, SCR, BUN, and TC [27]. In this study, we explored the protective effects of ergosterol on renal functional parameters and found that kidney index, urine volume, and the levels of urinary albuminuria, SCR, BUN, and TC were prominently increased in DN group, indicating that the DN model was successfully established and renal dysfunction occurred. However, ergosterol treatment prominently ameliorated those abnormal renal function parameters, suggesting that ergosterol exerted its reno-protective effects by improving functional parameters. The protective effect of ergosterol on renal function was further confirmed by the amelioration of histopathological injury. According to the histopathological results, we confirm that ergosterol notably inhibited glomerular hypertrophy, mesangial expansion, and collagen fiber deposition, suggesting that ergosterol may be an effective drug in the treatment of renal fibrosis associated with DN.

Over-proliferation of mesangial cells plays an important role in the progression of DN [28]. Mesangial cells could cause glomerular damage through the over proliferation of cells and the accumulation of ECM [29]. Consequently, it is now believed that the inhibition of mesangial cell proliferation is one of the targets that delays the progression of DN. In this study, expression of PCNA was used as a marker of over-proliferation. According to the MTT and Western blot analysis, we find that ergosterol prominently inhibited the hyperproliferation of mesangial cells induced by high glucose. These data indicate that ergosterol might relieve the renal fibrosis progression of DN.

Mesangial cells hyperproliferation caused an increase in ECM accumulation, which was consequently associated with eventual glomerulosclerosis and fibrosis [30]. Fibronectin and collagen I have been recognized as normal constituents of ECM and contribute to the incrassation of glomerular basement membrane and the deposition of ECM during the progression of DN [31,32]. MMPs are zinc-dependent proteolytic enzymes of the extracellular matrix which are involved in remodeling the ECM [33]. However, due to the complexity of MMP pathobiology, the activity of MMPs in DN described in various reports is not always consistent [8,34,35,36]. The contradiction in different works may be caused by the diversity in modeling methods, different phases of DN, and the complicacy of mechanisms responsible for DN development. In the present study, we found increased expression of fibronectin and collagen I and decreased expression of MMP-2 and MMP-9 in both STZ-injected mice and HG-treated mesangial cells. However, those variations were abrogated by ergosterol treatment. Those results indicate that ergosterol could mediate ECM accumulation in STZ-injected mice and HG-treated mesangial cells through the inhibition of ECM production and the acceleration of ECM degradation.

**Figure 9:**
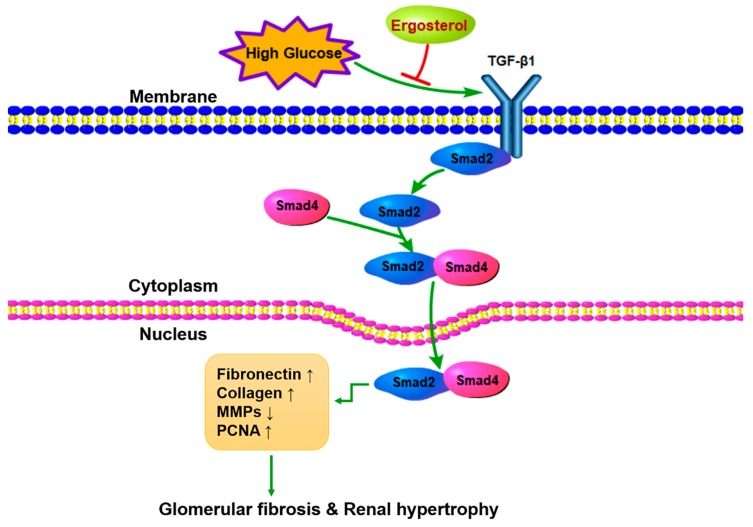
Schematic diagram shows the therapeutic effects of ergosterol on diabetic nephropathy.

TGF-β is reported to be elevated under diabetic conditions and plays an important role in the initiation and progression of DN [37]. TGF-β/Smads signaling is thought to be over-activated during DN and plays a prominent role in the proliferation of mesangial cells and in the deposition of ECM, which are the typical characteristics in early DN [38,39]. TGF-β1 activates the receptor-regulated Smad2, which interacts with Smad4 and translocates to the nucleus to regulate target gene expression [40]. Data from our research show significant activation of the GF-β1/Smad2 pathway in STZ-induced mice and HG-induced mesangial cells. Smad7, as an endogenous inhibitory Smad, negatively regulates the recruitment and phosphorylation of Smad2 [41]. In the fibrotic kidney, the expression of Smad7 is prominently decreased. In addition, ergosterol treatment was shown to decrease the phosphorylation of Smad2 and the expression of Smad4 protein as well as improving the expression of Smad7. Besides, we used a TGF-β1 inhibitor to validate the TGF-β1/Smad2 signaling pathway involved in the inhibiting effect of ergosterol on mesangial cell proliferation and ECM expression. LY2109761 is a small molecule inhibitor against TGF-β1, which could inhibit Smad2 protein phosphorylation [42]. Interestingly, there is synergy between LY2109761 and ergosterol in suppressing high glucose-induced mesangial cell proliferation and ECM expression. These results suggest that ergosterol negatively regulated the TGF-β1/Smad2 pathway to control renal fibrosis in DN (Figure 9).

## 5. Conclusions

In summary, the present work shows that ergosterol treatment alleviated metabolic parameters, renal pathophysiology, and ECM deposition, and inhibited renal TGF-β1/Smad2 signaling in STZ-induced diabetic mice. Additionally, high glucose activated TGF-β1/Smad2 signaling, thus leading to mesangial cell proliferation and ECM deposition in RMCs. However, ergosterol treatment strongly suppressed the TGF-β1/Smad2 signaling pathway, ultimately quenching the mesangial cell proliferation and ECM deposition induced by high glucose. These results suggest that ergosterol has a therapeutic effect in the prevention of diabetic nephropathy and is a promising therapeutic treatment to attenuate diabetic nephropathy.

## Figures and Tables

**Table 1:** Primer sequences used in real-time polymerase chain reaction (RT-PCR) analysis. MMP: matrix metalloproteinase.

Gene Name	Accession Number	Primer Sequence
Fibronectin	XM_017315848.1	Forward: 5′-GCCGAATGTAGATGAGGAG-3′ Reverse 5′-AGGAACTTGGAACTGTAAGG-3′
Collagen I	NM_007742.4	Forward: 5′-TGTGTGCGATGACGTGCAAT-3′ Reverse 5′-GGGTCCCTCGACTCCTACA-3′
MMP-2	NM_008610.3	Forward: 5′-TGGCAAGGTGTGGTGTGCGAC-3′ Reverse 5′-TCGGGGCCATCAGAGCTCCAG-3′
MMP-9	NM_013599.4	Forward: 5′-TCTTCGACTCCAGTAGACAATCCTT-3′ Reverse 5′-AATTGGCTTCCTCCGTGATTCG-3′
β-actin	NM_007393.5	Forward: 5′-TCCATCATGAAGTGTGACGT-3′ Reverse 5′-GAGCAATGATCTTGATCTTCAT-3′
